# A Qualitative Review of Community Health Workers’ Training, Supervision, and Service Delivery Needs

**DOI:** 10.1007/s10488-025-01439-w

**Published:** 2025-04-01

**Authors:** Maya Mroué Boustani, Stacy L. Frazier, Diana Marin, Dina Bashoura

**Affiliations:** 1 Loma Linda University, California, USA; 2 Florida International University, Miami, FL USA; 3 Department of Psychology, Loma Linda University, 11130 Anderson St., Suite 117, Loma Linda, CA 92350 USA

**Keywords:** Community health worker, Lay health workforce, Training, Supervision, Mental health

## Abstract

Community Health Workers (CHW) are part of an emerging workforce in the field of mental health, but few studies have examined their training, supervision, and service delivery needs in the United States. Individual semi-structured interviews were conducted with CHWs (n = 9) and their administrators (n = 6) affiliated with a medical center and school districts in Southern California. Guiding questions focused on CHW roles, work-related stress, clinical decision making, and professional development. Findings pointed to high rates of stress, primarily attributed to role ambiguity, and need (and desire) for more training and supervision related to mental health interventions, and more systematic structures for data-informed decision-making. Discussion provides reflections and recommendations to the field about ways to support this growing and critical workforce.

**Supplementary Information:**

The online version contains supplementary material available at 10.1007/s10488-025-01439-w.

## Introduction

Barriers to mental health care in under-resourced communities are well documented, including mental health workforce shortages and geographical misdistribution of providers (Barnett et al., 2016; Coker et al., 2009; Guerrero et al., 2019; Mongelli et al., 2020; Park et al., 2020; Sungkyu Lee et al., 2014), lack of insurance coverage (McAlpine & Mechanic, 2000), lack of community-based interventions (Stith et al., 2006), lack of diverse providers, and stigma (Knifton, 2012; Merwin et al., 2003). Geographic areas with a shortage of mental health workforce (Altschul et al., 2018; Andrilla et al., 2018), notably rural settings (Guerrero et al., 2019; Nayar et al., 2017) and areas of poverty (Kepley & Streeter, 2018), experience adverse outcomes, including higher rates of youth suicide (Johnson & Brookover, 2020).

Furthermore, lack of insurance coverage and community-based interventions disproportionately impact populations in under-resourced communities who, without insurance, cannot afford care (or, often, the time off work to attend appointments) and cannot access services that are unavailable in their communities and thus require additional time and travel (McAlpine & Mechanic, 2000). For those who receive care, quality is lower and often not evidence-based (Aisenberg, 2008; Alegría et al., 2008; Kenneth Wells et al., 2001). A lack of linguistically and culturally tailored services alongside rapidly changing demographics in the US (Huang et al., 2004) puts diverse populations (Black, Hispanic and Indigenous) at risk of not receiving services (Pumariega et al., 2005; Saechao et al., 2012). Mental health stigma is another key barrier that is prominent in under-resourced communities (Gary, 2005; Knifton, 2012; Nadeem et al., 2007). Expanding the mental health workforce, notably by training paraprofessionals such as Community Health Workers can be part of the solution to these barriers to care. However, the lack of clarity about the limitations of their roles, training and supervision needs limits their impact and puts them at risk for stress and burnout. This study sought to gather information to inform the field of CHW training, supervision, and service delivery support needs.

## Who are Community Health Workers (CHWs) and What Do They Do?

CHWs (also called health educators, promotores, lay health workers, paraprofessionals, and natural helpers) are a unique workforce. They have not completed post-baccalaureate clinical training but serve the communities to which they belong (Ayala et al., 2010; Viswanathan et al., 2010). CHWs are widely providing care in Low and Middle-Income Countries (LMIC) (Fulton et al., 2011), and are gaining increasing popularity in the US (Balcazar & George, 2018) because they reduce the cost of services and remediate barriers for under-resourced communities (Barnett et al., 2018; Gilkey et al., 2011; McQuillin et al., 2019; Rosenthal et al., 2010; Sabo et al., 2017). CHWs are both effective and cost-effective for chronic disease prevention and intervention in the US (Coker et al., 2009; Martin et al., 2018; Mongelli et al., 2020). Their effectiveness in addressing mental health needs has not yet been widely studied. In the most recent review of CHW-delivered mental health interventions, only ten took place in the US (Hoeft et al., 2018). Despite a lag in research, the workforce is rapidly growing. Although the exact number of CHWs in the US is difficult to estimate (due to varied labels describing them), the US Bureau of Labor Statistics reports 117,730 individuals with the work titles of “Community Health Worker” or “Health Educator”.

CHWs deliver a wide variety of care (e.g., diabetes lifestyle, prenatal care, prevention of school truancy, mental health first aid, etc.) in a wide variety of settings (e.g., schools, homes, hospitals, community-based organizations). As outlined by Berini and colleagues in their systematic review (2022), CHWs are sometimes direct providers of care (generally for low need and early intervention). At times, they simply coordinate care (help individuals set up appointments, pick up medication etc.). Malcarney and colleagues (2017) report in their systematic review that the roles of CHWs ranges from promotive to preventative and curative across human development. They provide prenatal support for new mothers, support for children, adolescents, adults and the elderly. They operate in a variety of settings. Some CHWs work directly in the healthcare settings, homes and communities.

CHWs can remediate several barriers to care (Mobula et al., 2015). First, CHWs can be trained in a short amount of time, reducing the pipeline from training to practice, and expanding the mental health workforce. Second, they can reach difficult-to-access communities (rural areas and areas with high poverty) and provide services in flexible environments (e.g., home or school visits) (Berini et al., 2022). Furthermore, they are more linguistically and culturally diverse than specialized providers, live in the communities they serve, and are considered credible and trustworthy based on their lived experiences (Ayala et al., 2010; Barnett et al., 2018).

Owing to the nature of their role as peer workers, there is little consensus or guidelines on training, supervision, and service-delivery. Generally, CHWs are trained by organizations that hire them, although certificate programs exist (Berini et al., 2022). Currently, there is no national consensus in the United States regarding certification or licensing for this workforce, although some states have made concerted efforts to provide structures around the roles of CHWs. This is also true in Canada and other high-income countries (Najafizada et al., 2015). For instance, where this study took place in California, CHWs are now officially recognized as providers of care by MediCal and Medicare, and their services are now billable (as of July 2022). Similarly, there is no consensus on whether and how much supervision is required. In an organizational chart, CHWs refer to care and coordinate care. Being a CHW tends to be a precarious job, usually hourly, and relies on grants, meaning that job security is not guaranteed. This has begun to change over the years as an increasing number of states recognize the important job that CHWs do and have begun to reimburse the services they provide (Scott et al., 2018). They are funded in a variety of ways ranging from federal, state, and local funds, including directly from health systems and health plans (Malcarney et al., 2017).

## CHWs Experience High Rates of Stress

Several unique complexities of their role put CHWs at risk for elevated stress. First, CHWs work with individuals who are highly stressed and exhibit multiple needs (Jacobs et al., 2017; Wennerstrom et al., 2011). As a result, their workload is high (Wennerstrom et al., 2011), and there may not be adequate time to provide care for everyone on their caseloads (Edwards et al., 2000). Prolonged exposure to work-related stress, such as holding high caseloads, can lead to burnout (Ducharme et al., 2007). Second, as aforementioned, CHWs live in the communities they serve (Ayala et al., 2010) and thus often experience similar stressors as their clients. They also have a deep connection to and understanding of their clients’ life stressors and circumstances, which puts CHWs at a higher risk for vicarious trauma (Thomson, 2014). Third, CHWs are likely to experience role ambiguity (Edwards et al., 2000) compared to traditional workforces who are more highly trained. They experience more discrepancies between their tasks and their skills. This imbalance between their resources (internal and external) and the reality on the ground (Jacobs et al., 2017) means that they are (and feel) underprepared to help their clients (von Hippel, 2019) and unclear about the boundaries of their roles (Lee et al., 2019). Finally, unlike licensed mental health professionals, CHWs lack a structure in their training, supervision, and service delivery protocols (Edwards et al., 2000).

## Training and Supervision Can Mitigate CHW Stress

A few studies encourage the use of individual interventions to reduce mental health staff stress and burnout (Morse et al., 2012); however, more research points to the need for an organizational approach (Burke & Richardsen, 2001; Stalker & Harvey, 2002), given that sources of stress are largely due to organizational factors such as large workloads, time pressures, role conflict or ambiguity, and lack of autonomy (Maslach et al., 2001; Paris & Hoge, 2010; van Dierendonck et al., 2001). Training and supervision can help mitigate burnout and increase resilience among paraprofessionals (Au et al., 2018; Scott et al., 2018). For instance, community mental health nurses with lower amounts of supervision exhibited higher rates of burnout than nurses with higher amounts of supervision (Edwards et al., 2006). Most CHW programs provide broad training in community outreach or advocacy (DePue et al., 2013), but limited training or supervision related to data-informed decision making, thus minimizing preparedness to deliver empirically supported mental health-promoting tools, despite serving patients with disproportionately elevated needs. Organizational support and structured protocols can mitigate stress and reduce turnover.

## Study Objectives

We interviewed CHWs and stakeholders to explore their training and supervision needs, and to inform the field of organizational strategies to reduce stress and burnout among paraprofessional mental health workers. These data serve as a starting point for the field and stakeholders to develop and implement comprehensive training and supervision models that meet CHWs’ professional needs and support their well-being. Semi-structured interviews were designed around guiding questions focused on CHW stress and burnout, including the role of training and supervision in their professional lives. Our qualitative approach was bottom-up, and themes emerged based on participants’ responses.

## Method

### Research Design Overview

#### Researcher Description

The Principal Investigators (first and last authors) of this study have a history of engaging in community partnerships and relying on qualitative methods to understand a community’s needs. Both of these researchers have supported workforces in under-resourced settings, including schools, community mental health, and after-school settings. The additional researchers are graduate students under the mentorship of the first author, who have an interest in supporting lay health workforces and underserved communities. The Principal Investigators’ backgrounds enhanced this research, given their deep understanding of workforce issues and their sensitive approach to community partnerships. The relative naivete of the interviewers balanced the PI’s expertise and allowed a more neutral interview experience for participants. 

### Participants

We recruited nine Community Health Workers (CHWs); and six stakeholders who work with CHWs (clinic directors, training directors, supervisors, administrators) from sites that train and employ CHWs in the San Bernardino area. This was a convenience sample, given that many of the recruited CHWs receive their certification and are contracted by the medical center where the researchers work (a large medical center east of Los Angeles).

CHWs identified as female (n = 6) and male (n = 3). Their ages ranged from 24 to 53 years, with a mean age of 40.5 (SD = 11.7). They identified as Hispanic or Latino (n = 7), and Black or African American (n = 2). Stakeholders identified as female (n = 4) and male (n = 2). Their mean age was 51.75 (SD = 14.45). They identified as Hispanic or Latino (n = 3), Black or African American (n = 1), Asian (n = 1) and White (n = 1). The CHWs we interviewed worked with youth in schools or with families at their homes in the city of San Bernardino, California and the surrounding area, an area with high levels of poverty and community violence. One group of CHWs worked specifically on school truancy. Their role was to visit the home of youth who were chronically absent from school in order to assess the reasons for the truancy and provide services and/or relevant referrals. The other groups of CHWs focused on mental health needs of youth in a neighboring district. In addition, although we requested to interview CHWs focused on behavioral health, we did get responses from three CHWs who focus on the needs of families experiencing healthcare related stressors: one CHW provides support for new mothers who’s babies had been discharged from the NICU, another was in charge of supporting families following a discharge from the trauma unit, and the last one was in charge of supporting families with a member who has diabetes. All of the CHWs supported families in the same community, an areas of Southern California experiencing high levels of poverty and community violence. All CHWs were certified. In order to achieve certification, CHWs attend classes by a partnering college that offers a CHW certification program. In order to qualify for certification, they had to have lived experience that aligns with and provides a connection with the community they were to serve. They could meet qualifications either through a certificate pathway, consisting of training and education. The certification program consisted of a core component focusing on individual and community capacity building, behavior change, cultural humility, health education and promotion, informal counseling skills, and advocacy. Thereafter, based on their interest, they can select a behavioral health track or clinic-based track. The CHWs who participated in this interview were behavioral health focused and took courses in the mental health system, common mental health disorders, risk assessment and basic crisis response, Motivational Interviewing and Mental Health First Aid. The training could be completed in six months via two days per week or in 10 weeks via three days per week training. Alternatively, they could qualify via the work experience pathway, by documenting 2000 h of work serving as a CHW (either paid or volunteer) within the past three years. All CHWs must complete six hours of continuing education yearly.

The stakeholders consisted of two supervisors of CHWs, who provide direct supervision to the CHWs we interviewed, one administrator of CHW contracts, two administrators of CHW training programming, and one school principal which housed an active CHW program for school absenteeism. These stakeholders were all part of the network within our institution and worked either directly (supervisors) or indirectly (administrators and directors) with the CHW participants. The interview questions were the same as those posed to the CHWs (replaced “you” with “CHWs at your institution”). We used the same procedures and interviewers as for the CHWs.

## Researcher-Participant Relationship

The first author and graduate students are part of the same institution as the one that contracts CHWs to work in communities. However, the researchers are part of the Department of Psychology at the institution. The participants are employed by a different entity on campus that supports the community surrounding the university. This entity reached out to the first author to engage in a partnership to support the CHWs. This facilitated our research efforts while maintaining an appropriate separation, as the researchers do not know or interact with the CHWs outside of the research setting.

## Participants Recruitment

### Recruitment Process and Participant Selection

CHWs and stakeholders were recruited from an institute that employs CHWs and contracts them to work in various settings (schools, community mental health, hospitals, etc.) in Southern California. Most of these CHWs are certified and work full-time in school, hospital, and community settings supporting youth and family mental health.

The institute’s administrator distributed an announcement via email to CHWs about the opportunity to participate in research to better understand CHW’s needs for training and supervision. The research team then attended a CHW supervision meeting to explain the purpose of the study, and the administrator sent a follow-up email with a link to an online survey, should they wish to participate. Interested participants signed a digital consent form, completed a demographic information survey, and provided contact information to schedule a semi-structured interview. A total of 15 CHWs received the email, with a total of nine agreeing to be interviewed– we interviewed all interested CHWs. We reached out to all the stakeholders in our network via email– they all agreed to participate. Our goal was to interview 10 to 15 participants, in order to achieve saturation during our interviews, as recommended by Hennink & Kaiser (2022) and as was achieved by our team in the past. The CHWs and stakeholders were offered a $25 gift card as a token of appreciation for their participation. All procedures were approved by our Institutional Review Board.

## Data Collection

Data were collected through individual semi-structured interviews. Two graduate students received a 90-min training on how to conduct semi-structured interviews, including interview techniques (using open-ended questions, avoiding bias, building rapport) and engaged in role plays with an interview guide developed for this study. The training was delivered by a researcher (the first author) with extensive experience in qualitative methods. Each graduate student interviewed four to five participants, which took approximately one month to complete. Interviews were conducted via video conferencing or phone with just the interviewee and the interviewer present. During the hour-long interviews, the participants were reminded of their right to stop the interview and the recording at any time if they chose to do so. An interview guide was used to guide the research assistants through open-ended questions. For the purpose of this study we were interested in questions about CHW roles, sources of work-related stress, training, and supervision. Additionally, we were interested in understanding how CHWs engage in clinical decision-making, so we asked, “How do CHWs know which intervention to use for what problems?”. The interview guide also included questions about typical problems encountered among the families they served. Interviewers were free to probe for more information, as appropriate, based on participants’ responses to guiding questions. The interviews lasted between 37 to 127 min, with an average interview time of 60 min.

## Recording and Data Transformation

All interviews were audio-recorded and transcribed automatically using a transcription software. All the transcripts were de-identified, checked for accuracy, and coded. All audio files were deleted after the completion of transcriptions.

## Analysis

### Data Analytic Strategies

The research team followed qualitative analysis procedures based on thematic analysis, as outlined by Braun & Clarke (2006). Four coders were recruited for the study. Coder 1 and Coder 2 consisted of the Principal Investigator (a licensed clinical psychologist and Assistant Professor) and a graduate student with prior experience conducting qualitative interviews and working in communities. They independently engaged in *open coding*, gathering keywords or phrases by reviewing all of the qualitative interview transcripts. Both coders then met and compared codes for similarities and differences, ensuring that all the necessary categories were included. Following the open coding process, *axial coding* involved having the same two coders individually make connections between categories, identifying ways they relate to each other. Codes were created to capture CHWs job descriptions, stressors, problems encountered, training models, supervision models, and service-delivery decision-making models. They were organized into a codebook with sub-codes, definitions, and examples of how the codes may be applied. Coders 1 and 2 then tested the codebook on separate transcripts and adjusted codes, as needed, to confirm that the codebook was ready for use. Two new naïve coders (coders 3 and 4) participated in codebook training where they became familiar with all codes. Coders 3 and 4 were doctoral students in clinical psychology with extensive experiencing working in communities. Coder 3 worked with CHWs in international settings previously, while coder 4 had experience working with youth and staff in foster care systems. Before coding began, all transcripts were segmented into excerpts by Coders 1 and 2. Excerpts varied in length from one sentence to one paragraph. An excerpt was identified as a unit of analysis, such that the content was a response to a topic and part of a theme that provided context to understand a response. An excerpt can include one or multiple codes, depending on its content. Excerpting the transcripts ahead of time facilitates consensus coding and calculations of reliability estimates. Coders 3 and 4 first coded a practice transcript together, and then another practice transcript independently. Once they became comfortable with all the codes, they each coded the same transcripts independently, such that all transcripts were double-coded. Qualitative coding was performed using Dedoose (2021), a qualitative coding software package. After all the transcripts were successfully coded, frequency counts for each code were calculated using Dedoose to determine the percentage of CHWs and stakeholders that endorsed different themes regarding CHW roles, training, supervision, service delivery, and problems encountered. In addition, the coders were encouraged to identify quotes that they felt best represented each code.

## Methodological Integrity

All the transcripts were double-coded. Coders 3 and 4 engaged in consensus coding via weekly meetings with Coders 1 and 2 to moderate the discussion and settle inconsistencies in cases of disagreement. If coders were unable to settle their disagreement, Coder 1 (the PI) would then consult the transcript and determine which code best matches the excerpt in question. Weekly meetings to discuss any challenges and review codes continued through the end of the coding to prevent coder drift. Reliability estimates between coders (prior to consensus meetings) was satisfactory, with Cohen’s Kappas ranging from 0.29 to 0.97, with a mean of 0.59.

## Results

We describe frequency counts in percentages representing the number of excerpts (single unit of analysis) identified for each code under a particular category. We coded 15 transcripts, capturing 872 excerpts, with an average of 58 excerpts per transcript. These codes are organized into five umbrella categories: (1) Roles, (2) Stressors, (3) Training, (4) Supervision, (5) Problems Encountered, and (6) Service Delivery. As such, when reporting a percentage, we are referring to the percentage of excerpts for which a given code was applied in that category (percentage of excerpts). The total percentages may add up to more than 100, as more than one code may be applied to a single excerpt. We also report the percentage of participants who endorsed each particular code at least once (percentage of participants). The total percentages may add up to more than 100, as each participant may endorse more than one code for each category. We report subcodes that are mentioned in 10% or more of the excerpts.

## CHW Roles (n = 262 excerpts)

Participants provided descriptions of CHW roles related to job demands and clarity of expectations. All participants (100%) described CHW’s roles revolving around some form of community outreach (69% of excerpts), such as screening for symptoms and providing referrals. For example, one CHW described simply providing shelter referrals for the homeless: “*I've been in contact with at least over 100 homeless patients, okay. I refer each and every one of them to a shelter.*” (CHW#104).

Several participants (73%) described CHWs as also providing auxiliary care (23% of excerpts) such as psychoeducation and promoting treatment engagement by addressing barriers to care, such as* “… a lot of behavioral health tips (…) teaching them how to reach out and get resources*” (CHW #112). Another 11% of excerpts (endorsed by 40% of participants) described their role as providing a stepped care approach to intervention, such as screening for symptoms, offering simple prevention tools or treatment recommendations and referring to professional providers for more intense services when this seemed warranted. For instance, “*micro interventions of anything like on the moment just to maybe stabilize the situation and then [a] referral*” (CHW #111). Only 1% of excerpts (endorsed by just one CHW, 6%) described CHWs as primary providers of services, reflecting, for example, the following message that was regularly reinforced by administrators:

*“But this is where you have to draw the line, you are not a clinician, and we talk about that during the training all the time. Remember, you are not a clinician, you may come to our training with an MA, or even RN degree from your country, but here in your job, under, you know, in United States, you're not, your job is not a clinical responsibility, it is to serve as a connector liaison, as a frontline worker*” (Stakeholder #113).

Despite being able to describe their official role, several CHWs reported that their roles and responsibilities were unclear, leading to stress and uncertainty. Some CHWs who immigrated from other countries had higher credentials that were not recognized in the United States. A lack of licensing or regulatory boards further complicates care delivery. As one stakeholder described:

“*The CHW practice is not as clearly defined as some of the [other] clinical practices, especially in California because we don't have a statewide or state level certification or licensure. And there's such a broad wide spectrum of (…) scope of practice that CHWs do from the very grassroots to the very integrated complex systems*” (Stakeholder #113).

When reflecting on their ability to document services in the agency’s electronic medical record, one CHW expressed frustration at not being considered or recognized as a provider, despite their significant workload and contribution to care:

*“The only thing is that we still [cannot do], we can still have a signature in there, because we are not providers, we not considered as a provider, which I think we are really, which I think we are we are kind of other providers, we're still providing. We can, like I said before, we are not therapists, we can help have a therapeutic session. So that's different. And we provide some type of services. I understand that the health system has been like this like for life for many years. But I really hope that in the future and the health system can see us as providers too. And we can actually be able to sign our documentation without having a cosign, and they can see, okay, this is the evaluation, the work, treatment, and the result for this CHW working with this patient*” (CHW #102).

## CHW Stressors (n = 189 excerpts)

The CHWs and stakeholders described several sources of stress, including administrative requirements (20% of excerpts endorsed by 60% of participants), clarity on role limitations (14% of excerpts endorsed by 60% of participants), personal stressors (12% of excerpts endorsed by 47% of participants), feeling overworked (9% of excerpts endorsed by 47% of participants), and experiencing secondary trauma from their cases (6% of excerpts endorsed by 40% of participants). One stakeholder described stressors surrounding role ambiguity as follows:

“*… trying to keep them in that narrow scope of work: it is your job is to refer and identify, but not to treat not to prescribe. And then staying within those boundaries, I think they find the difficulty in the handoff and then that you know produces undue stress for them, especially if a provider is not responding to their patient or participant in the program” (*Stakeholder # 114).

A recurring theme reflected that CHWs felt obligated to attempt to resolve their clients’ issues while being told by their leadership that it was not their job. As the same stakeholder put it:

*“… even if it's [not] explicitly said like your job is to link and refer families, and then you you're doing a handoff, they still want to go further and feel like they have to have all the knowledge for everything. That a family member is wanting or student needs. But reminding them that you're, there's no one person that can hold all this knowledge. We just have to know the people who do. So, I think they take on that burden of I have to know and do everything.”* (Stakeholder # 114).

## CHW Training (n = 186 excerpts)

CHWs and stakeholders stated that the training that they receive emphasizes mental health (39% of excerpts endorsed by 100% of participants), school support (16% of excerpts endorsed by 60% of participants), and transition support (12% of excerpts endorsed by 53% of participants). CHWs often expressed the desire and need for mental health training to better equip themselves for the needs of the people they serve.

*“if we had our choice, we would maybe expand on the mental health component. It seems to be a high need, you know, there seems to be a lot of needs when it comes to mental health”* (CHW #112).

Other codes identified under the theme of training included medical training (10% of excerpts endorsed by 100% of participants), referring largely to medical terminology (e.g., HIPAA) and procedures (e.g., health insurance, resources, and referrals). Finally, some participants referred to the need for active learning strategies (9% of excerpts, endorsed by 33% of participants), with several participants referring to the importance of relying on role-playing as an active training strategy. As one CHW described:

“*I enjoy the hands on I really would appreciate it more hands on and role playing you know, the role playing was very, very important. And I wish we would have had more time for that”*(CHW # 103).

## CHW Supervision (n = 119 excerpts)

Four codes emerged to describe the routines of CHW supervision. Most often, participants described reaching out to their supervisors for hands-on support with cases (24% of excerpts, endorsed by 60% of participants), reflecting back to the stress surrounding role ambiguity:

“*So, then I would go to [supervisor’s name] and I'm like, What do I do? What do you think? Like, I ask her a lot. I don't want to step over any boundaries. I don't want us to get into any type of trouble. So (…) I'm always asking her..*.” (CHW #101).

Supervision style was also represented in 24% of excerpts and endorsed by 87% of participants. Here, they describe their supervisor’s general style, such as:

“*She's also very, very caring for like the time She knows we all have family. She knows. We have challenges here in the clinic and (…) she is very flexible. She’s very thoughtful also*” (CHW #102).

Supervision was described as an opportunity to receive professional development (18% of excerpts endorsed by 67% of participants). CHWs appreciated this extra opportunity for training. As one stakeholder described:

“*So we were having monthly professional development meetings (…) one will be on coping skills for the work that they have and avoiding burnout. And then we're just setting up a series every month to be consistent now, so that we can continue to professionally develop them”* (Stakeholder #111).

Finally, supervision presented an opportunity to address administrative issues (15% of excerpts endorsed by 67% of participants) such as documentation and tracking:

“*we would go over like paperwork or if we had a question about anything that we needed to complete, then we could talk to her about right there and then. if we had any general questions, then we could ask her like this especially about like cases, if you have 20 cases or cases completed, what home visit number are you on, things like that? So, I think it was, it was good to keep us accountable and to keep us up to date and you know, if they're on the third you need to complete this and if they're on your fourth thing you need to turn in this*” (CHW #105).

While the CHWs were overall satisfied with their supervision experience, they nonetheless felt like they could use more hands-on support and guidance on how to manage the problems they encountered. For instance, one CHW shared that having live supervision would be helpful:

“*Like, doing the home visits live and should should write down notes, like, kinda like you taking your driver's test. And then at the end, she's gonna be like, oh, I've noticed this. I noticed the way you talk, your gestures*” (CHW 108).

They also desired more communication about cases, citing a lack of supervisors’ understanding of the realities on the ground:

*“And I've noticed that maybe sometimes lack of communication, she might feel like the CHWs are pushing back on her. But in reality, it's just like, that's just a way of like, giving suggestions because as community health workers, we do see a lot of things that could work. And sometimes you want to share that with the supervisor, but it's hard for them to, to understand, because they're not out in the community”* (CHW #108).

## CHW Problems Encountered (n = 109 excerpts)

Seven codes represented the types of problems CHWs encountered when serving families. Given that we recruited CHWs delivering behavioral health, it is not surprising that the most commonly encountered problem reported revolved around mental health concerns (36% of excerpts, endorsed by 67% of participants) with another 13% of excerpts, endorsed by 33% of participants, focusing specifically on disruptive behaviors. As one participant noted:

“*I would say cause the students are going through a lot. I mean, it's more than my scope of work. I've had one student who was bipolar with a, he would hallucinate (…) Some have bad depressive symptoms that I see and that they say that they don't feel right, they feel sad all the time.*” (CHW # 105)

Access to basic needs such as shelter and food was endorsed by 73% of participants and constituted 29% of excerpts. CHWs referred to financial stressors that impacted the whole family– highlighting how this leads to feelings of stress among the CHWs as well:

“*You know, sometimes these families experience economic difficulties where I almost want to say, here is some money so you don't get kicked out of your home, or so you can pay your gas on the past due account. It happened to me to almost have to give some money to a family so they can pay the rent or gas account.*” (CHW # 107)

Additional problems encountered included domestic violence (6% of excerpts, endorsed by 27% of participants), and chronic health problems (6% of excerpts, endorsed by 33% of participants).

## CHW Service Delivery (n = 72 excerpts)

Three codes represented how CHWs make decisions about which interventions to deliver for which problems. Structured decision-making practices were highlighted in 50% of excerpts (endorsed by 80% of participants), compared to 24% emphasizing unstructured decision-making (endorsed by 73% of participants). The theme of confidence or lack of confidence emerged 24% of the time among 60% of participants. In particular, both stakeholders and CHWs expressed that it would be helpful to have structured tools, such as decision-making flowcharts, to assist them. As one stakeholder outlined:

*“Maybe having kind of a map that says When this happens, that's what you do. I can be I guess that's what it would be a protocol that would exist out there where they would have this big picture. Showing when this circumstance happened, that's what you do. If it's a yes, you do that it is a no you do the other. So that might be something that if it was in place, if someone had developed that, it would help, especially the ones who just who are just starting*” (Stakeholder #113).

## Differences Between Stakeholder and CHW Responses

We asked CHWs and stakeholders the same questions during interviews (rephrased as needed, see appendix for copies of the interview scripts). When observing frequency counts of the parent codes, the main differences that emerged between CHWs and stakeholders were around the amount of details provided within certain codes. For instance, CHWs were more descriptive when discussing the problems they encounter during their work while stakeholders were more descriptive when discussing potential decision-making support tools to support CHWs. Both groups had similar intensity in discussions around supervision and training needs, indicating common goals.

## Overview of Themes That Summarize Codes

**Fig. 1:**
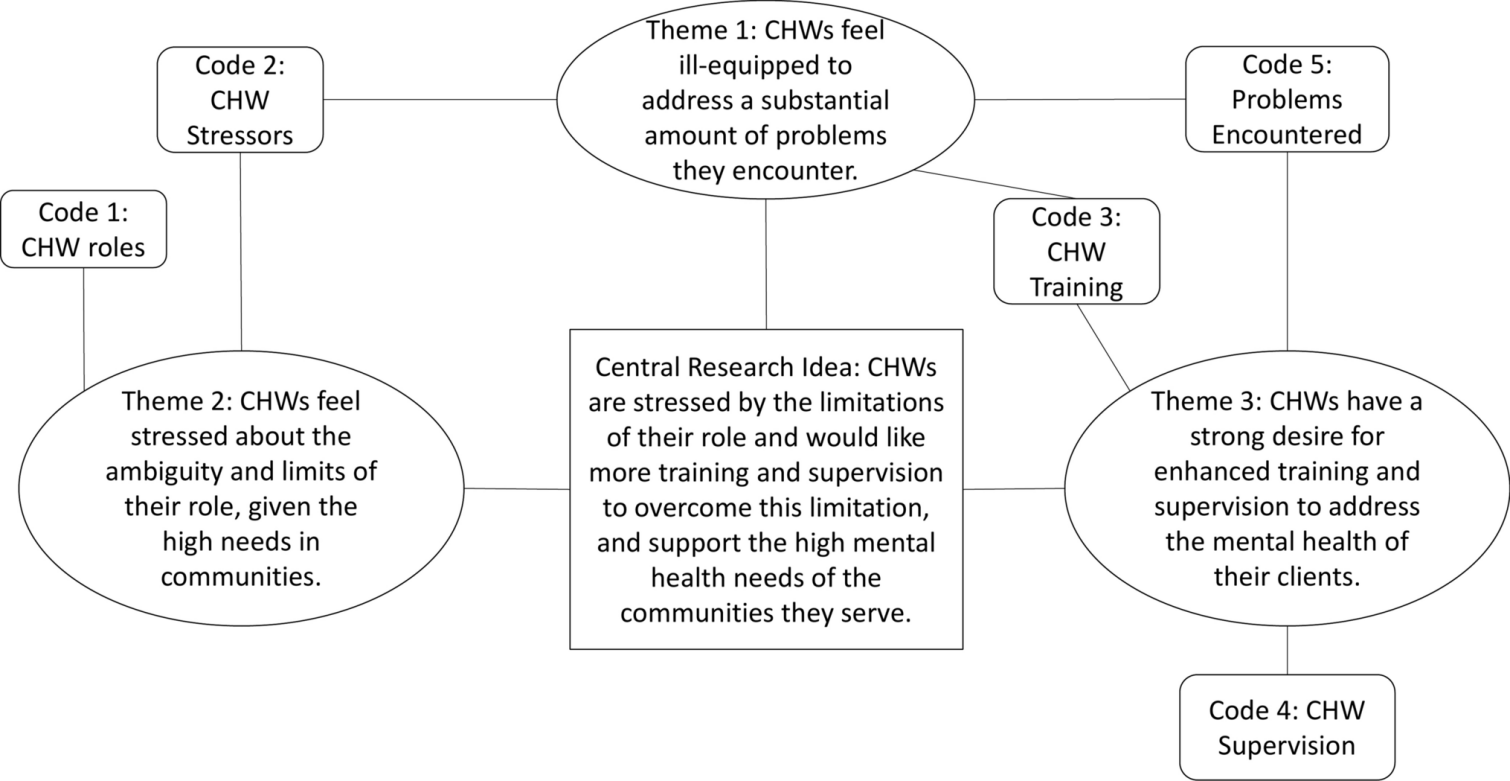
Relationship between codes

The relationship between the codes can be summarized around three themes: (1) CHWs feel ill-equipped to manage some of the problems they encounter in the community; (2) CHWs feel stressed around the ambiguity and limits of their role; and (3) CHWs have a strong desire for enhanced training and supervision to better address the problems they encounter. We summarize these in Fig. 1.

## Discussion

We explored the perceptions of CHWs and their administrators around job demands and sources of stress toward informing a workforce enhancement model for training, supervision, and service delivery. Clarity around the limitations of their roles emerged as a top stressor, and participants’ expressed interest was high for more training. The CHWs reported that their current model of supervision includes opportunities for check-ins, professional development, and peer support. Our findings indicate that CHWs want tools to help them make informed decisions regarding care delivery. These are important for contextualizing and planning workforce enhancement through training and supervision.

The CHWs and stakeholders in our study reported high levels and multiple sources of stress, ranging from personal to administrative. These stress levels are consistent with those reported in the literature on CHW stress in LMICs (e.g., Pakistan (Haq et al., 2008), China (Li et al., 2014), Iran (Bijari & Abassi, 2016), Ethiopia (Selamu et al., 2017) and other helping professions (eg., community mental health nurses (Edwards et al., 2000; Hannigan et al., 2000); care coordinators (Au et al., 2018); mental health workforces (Morse et al., 2012; Onyett et al., 1997; Paris & Hoge, 2010; Rössler, 2012; Wilson, 2016); educators (Gluschkoff et al., 2016; Moate et al., 2016); and healthcare workers (Cohen-Katz et al., 2005; Tijdink et al., 2014).To address high stress, CHWs may benefit from the tools they learn to deliver (Atanes et al., 2015; Au et al., 2018; Edwards et al., 2000; Hannigan et al., 2000; Morse et al., 2012). For instance, brief training for providers in problem-solving and relaxation has been demonstrated to reduce their own stress, mitigate burnout, build resilience, and improve their mental health (Wennerstrom et al., 2011). Programs that address stress and burnout directly use training in psychosocial interventions as a way to improve attitudes about behavioral interventions and increase provider self-efficacy to reduce stress and burnout (Corrigan et al., 1997; Ewers et al., 2002). Others introduce cognitive behavioral strategies to staff (relaxation, cognitive restructuring) and train supervisors in using these methods, resulting in reduced burnout rates among direct mental health care staff (Scarnera et al., 2009; Van Dierendonck et al., 1998). Mindfulness-based interventions have also been beneficial in decreasing stress and emotional exhaustion (a component of burnout) among paraprofessionals from diverse backgrounds working in underserved communities (Jacobs et al., 2017).

CHWs reported that one reason for their stress was their inability to address some of the presenting problems of the communities they serve. They report wanting enhanced training in mental health content to help them with that. In addition, they report that having supervision around how to move forward with a case (knowing when to refer out versus when to intervene and what tool to use when intervening) would relieve the stress they feel due to some ambiguity around their role. Role ambiguity is defined as a lack of clarity in understanding actions to be taken to achieve work goals (Kahn et al., 1964) and has been linked to poor job satisfaction and burnout in service professions generally (Mañas et al., 2018) and among peer support specialists (Abraham et al., 2021). In particular, CHWs were frustrated by the limits of the care they could provide versus the high needs of the communities they serve. On the one hand, they serve the most vulnerable and marginalized communities. On the other hand, their ability to provide EBPs is routinely under-estimated (despite CHWs in LMICs doing so routinely) and, thus, they are not systematically receiving supervision tools and training beyond outreach and auxiliary care. Instead, CHWs reported that they consistently receive the message that they are not clinicians and are not expected (or officially permitted) to provide any clinical care. Nevertheless, CHWs find themselves interacting with individuals at some critical moments, and they may benefit from being trained in robust transdiagnostic mental health tools that can be shared with those they serve, while awaiting more formal evaluation and services by an overburdened system.

We recommend reducing role ambiguity by training CHWs in evidence-based practices to address the most pressing needs of their communities and providing them with the supervision support to do so effectively and efficiently. At this critical time of elevated and disproportionate mental health needs in the United States, CHWs represent a capable and compassionate but under-trained and under-utilized workforce for delivering evidence-informed care. This may be driven by a lack of licensing and regulations regarding CHW roles. Notably, while a licensing board may help to structure training around specific competencies and increase clarity around role limitations, it may also discourage individuals from joining the profession by creating additional barriers (testing, paperwork, license renewals, fees, etc.).

## Strengths and Limitations

We combined stakeholders and CHWs in this sample. While this provided us with rich information from different perspectives, our sample size was too small to allow for separate analyses of stakeholders versus CHWs to compare and contrast their feedback. Furthermore, this study is limited to a small group of CHWs and stakeholders in one location in the United States. Therefore, these results should be interpreted with caution and may lack generalizability. Nevertheless, as California is transitioning to a model of reimbursing care delivered by CHWs, we believe that speaking to CHWs directly as well as the stakeholders who work with them is a strength.

## Future Directions

Our work with CHWs and other lay health workforces, along with the findings from this and other studies lead us to make some suggestions for future studies around training, supervision and service-delivery models for CHWs that can optimize the effectiveness, efficiency, and satisfaction of CHWs (Lyon & Koerner, 2016; Lyon et al., 2019).

*Screening and progress monitoring* have gained traction among licensed providers (Dyer et al., 2016), but the literature on CHWs and paraprofessional workforces is limited. Screening and monitoring identify problem areas, estimate symptom severity, and highlight improvement or deterioration over time to inform treatment planning. Routine monitoring improves clinical care and reduces client symptoms, as outlined in Feedback Intervention Theory (Kluger & DeNisi, 1996). Based on literature, and aligned with CHWs’ work in school-, hospital- and community settings, we recommend screening and progress monitoring with tools that are reliable, valid (Becker-Haimes et al., 2020), brief, easy to administer, generalizable across age groups, settings and problem areas (Glasgow et al., 2014), and effective (Newman, 2000; Otto et al., 2000).

*Common Elements* are mental health-promoting strategies (elements) common to evidence-based treatments within and across problems, outcomes, or contexts (Boustani et al., 2017a, 2017b). Relevant to the work conducted by CHWs, the common elements of mental health prevention and promotion programming may be useful (Boustani et al., 2015, 2020a, 2020b; Rith-Najarian et al., 2019) for children, adolescents, and young adults (Boustani et al., 2017a, 2017b; Boustani, 2020a; Chorpita et al., 2020; Cappella et al., 2008; Frazier et al., 2019; Frazier et al., 2015; Hedemann & Frazier, 2017; Michelson et al., 2019; Rith-Najarian, 2019; Shernoff et al., 2011). Several of the most common elements (e.g., problem-solving, communication skills, cognitive coping, and relaxation*)* align with CHW targets and expressed interests of school paraprofessionals (Riggs, 2001). Training CHWs to deliver such transdiagnostic elements will allow them to provide brief, evidence-informed, and supportive strategies to their clients while they wait for higher levels of care if needed. These elements can be helpful across settings, levels of care, and age groups– as demonstrated by their presence across these domains. We recommend relying on these single elements as key training and service delivery strategies to support as many community members as possible.

*Data-informed decision-making tools* can help CHWs make decisions (when to refer, when to intervene, using what tool, etc.). In this study, CHWs expressed interest in such data-informed decision tools. Measurement feedback systems are linked to faster progress (Bickman et al., 2011), better engagement and adherence (Lambert & Shimokawa, 2011), and reduced premature dropout (Bickman et al., 2011; Lambert et al., 2003) in community mental health care. They have been examined in a small number of paraprofessional studies including in community health centers for diabetes care (Rodriguez et al., 2018), clinics for prenatal HIV Prevention (Bull et al., 2018), and clinics for childhood illnesses (Roberton et al., 2016). Evidence of its acceptability comes from a post-Katrina study in New Orleans, in which CHWs expressed that screening and referral resources were among the most helpful in their program to inform care and referral (Wennerstrom et al., 2011). Data-informed decision tools are particularly applicable to CHW-delivered mental health services because they are cost-effective (many valid screeners are available for free) (Becker-Haimes et al., 2020), easily scalable (Borntrager & Lyon, 2015), efficient, and brief (Lambert, 2012). We believe that such solutions are timely as states begin to authorize the reimbursement of mental health prevention services delivered by CHWs.

## Conclusion

Findings offer insight into the training and supervision needs of lay health workforces. The mental health workforce shortage in the United States is projected to worsen (Kepley & Streeter, 2018), and a new generation of mental health models and workforces is needed to mitigate the risk of mental health problems and expand equitable access to quality care. As Community Health Workers play an increasing role in supporting community mental health in the US, future research will be necessary to design and examine the most efficient and effective models for workforce support, with an eye toward maximizing strengths and minimizing stressors.

Structured training, supervision, and data-informed decision tools for care and referral will help ensure that CHWs are an effective and efficient workforce that is well-equipped and well-positioned to reduce barriers to care and disparities for under-resourced and systemically marginalized communities. CHW support for stress management will ensure that this workforce is well-supported and retained. Based on the findings from this needs assessment, we further recommend that stakeholders who engage with CHWs be mindful of stressors that may impact them. We advise providing opportunities for self-care as part of structured supervision that takes place at regular intervals. We encourage future researchers to test models of training, supervision and care delivery that work for the contexts in which CHWs deliver care and, if possible, study those systems along with other outcomes of interest. Brief qualitative interviews with CHWs (whether in a clinical or research context) can provide insight to stakeholders around areas of improvement.

## Supplementary Information

Below is the link to the electronic supplementary material.

Supplementary file1 (PDF 158 kb)

Supplementary file2 (PDF 165 kb)

## References

[CR1] Abraham, K. M., Erickson, P. S., Sata, M. J., & Lewis, S. B. (2021). Job satisfaction and burnout among peer support specialists: The contributions of supervisory mentorship, recovery-oriented workplaces, and role clarity. *Advances in Mental Health,**20*(1), 38–50. 10.1080/18387357.2021.1977667

[CR2] Aisenberg, E. (2008). Evidence-based practice in mental health care to ethnic minority communities: Has its practice fallen short of its evidence? *Social Work,**53*(4), 297–306. 18853666 10.1093/sw/53.4.297

[CR3] Alegría, M., Chatterji, P., Wells, K., Cao, Z., Chen, C. N., Takeuchi, D., Jackson, J., & Meng, X. L. (2008). Disparity in depression treatment among racial and ethnic minority populations in the United States. *Psychiatric Services,**59*(11), 1264–1272. 10.1176/appi.ps.59.11.1264 18971402 10.1176/appi.ps.59.11.1264 PMC2668139

[CR4] Altschul, D. B., Bonham, C. A., Faulkner, M. J., Pearson, A. W. F., Reno, J., Lindstrom, W., & Larson, R. (2018). State legislative approach to enumerating behavioral health workforce shortages: Lessons learned in New Mexico. *American Journal of Preventive Medicine,**54*(6), S220–S229. 10.1016/j.amepre.2018.02.005 29779546 10.1016/j.amepre.2018.02.005

[CR5] Andrilla, C. H. A., Patterson, D. G., Garberson, L. A., Coulthard, C., & Larson, E. H. (2018). Geographic Variation in the Supply of Selected Behavioral Health Providers. *American Journal of Preventive Medicine,**54*(6 Suppl 3), S199-s207. 10.1016/j.amepre.2018.01.004 29779543 10.1016/j.amepre.2018.01.004

[CR6] Atanes, A. C., Andreoni, S., Hirayama, M. S., Montero-Marin, J., Barros, V. V., Ronzani, T. M., Kozasa, E. H., Soler, J., Cebolla, A., Garcia-Campayo, J., & Demarzo, M. M. (2015). Mindfulness, perceived stress, and subjective well-being: A correlational study in primary care health professionals. *BMC Complementary and Alternative Medicine,**15*, 303. 10.1186/s12906-015-0823-0 26329810 10.1186/s12906-015-0823-0 PMC4557919

[CR7] Au, M., Kehn, M., Ireys, H., Blyler, C., & Brown, J. (2018). Care coordinators in integrated care: Burnout risk, perceived supports, and job satisfaction. *American journal of preventive medicine,**54*(6), S250–S257. 10.1016/j.amepre.2018.01.044 29779549 10.1016/j.amepre.2018.01.044

[CR9] Ayala, G. X., Vaz, L., Earp, J. A., Elder, J. P., & Cherrington, A. (2010). Outcome effectiveness of the lay health advisor model among Latinos in the United States: An examination by role. *Health Education Research,**25*(5), 815–840. 10.1093/her/cyq035 20603384 10.1093/her/cyq035 PMC2948840

[CR10] Balcazar, H., & George, S. (2018). Community health workers: Bringing a new era of systems change to stimulate investments in health care for vulnerable US populations. *American journal of public health,**108*(6), 720. 29741936 10.2105/AJPH.2018.304427 PMC5944897

[CR11] Barnett, M. L., Davis, E. M., Callejas, L. M., White, J. V., Acevedo-Polakovich, I. D., Niec, L. N., & Jent, J. F. (2016). The development and evaluation of a natural helpers’ training program to increase the engagement of urban, Latina/o families in parent-child interaction therapy. *Children and Youth Services Review,**65*, 17–25. 10.1016/j.childyouth.2016.03.016

[CR12] Barnett, M. L., Gonzalez, A., Miranda, J., Chavira, D. A., & Lau, A. S. (2018). Mobilizing Community Health Workers to Address Mental Health Disparities for Underserved Populations: A Systematic Review. *Administration and Policy in Mental Health and Mental Health Services Research,**45*(2), 195–211. 10.1007/s10488-017-0815-0 28730278 10.1007/s10488-017-0815-0 PMC5803443

[CR13] Bashoura, D., Marin, D., & Boustani, M. (2020). *A Needs Assessment of Community Health Workers who work with Underserved Children and Families in the United States.* 54th Annual Convention of the Association of Behavioral and Cognitive Therapies

[CR14] Becker-Haimes, E. M., Tabachnick, A. R., Last, B. S., Stewart, R. E., Hasan-Granier, A., & Beidas, R. S. (2020). Evidence Base Update for Brief, Free, and Accessible Youth Mental Health Measures. *Journal of Clinical Child & Adolescent Psychology,**49*(1), 1–17. 10.1080/15374416.2019.1689824 31825683 10.1080/15374416.2019.1689824 PMC6962529

[CR15] Berini, C. R., Bonilha, H. S., & Simpson, A. N. (2022). Impact of Community Health Workers on Access to Care for Rural Populations in the United States: A Systematic Review. *Journal of Community Health,**47*(3), 539–553. 10.1007/s10900-021-01052-6 34817755 10.1007/s10900-021-01052-6

[CR16] Bickman, L., Kelley, S. D., Breda, C., de Andrade, A. R., & Riemer, M. (2011). Effects of routine feedback to clinicians on mental health outcomes of youths: Results of a randomized trial. *Psychiatric Services,**62*(12), 1423–1429. 10.1176/appi.ps.002052011 22193788 10.1176/appi.ps.002052011

[CR17] Bijari, B., & Abassi, A. (2016). Prevalence of Burnout Syndrome and Associated Factors Among Rural Health Workers (Behvarzes) in South Khorasan. *Iranian Red Crescent Medical Journal,**18*(10), e25390–e25390. 10.5812/ircmj.25390 28180014 10.5812/ircmj.25390 PMC5286445

[CR18] Black, N. (2013). Patient reported outcome measures could help transform healthcare. *BMJ,**346*, f167. 10.1136/bmj.f167 23358487 10.1136/bmj.f167

[CR19] Borntrager, C., & Lyon, A. R. (2015). Client progress monitoring and feedback in school-based mental health. *Cognitive and behavioral practice,**22*(1), 74–86. 10.1016/j.cbpra.2014.03.007 26257508 10.1016/j.cbpra.2014.03.007 PMC4524776

[CR20] Boustani, M. M., Frazier, S. L., Becker, K. D., Bechor, M., Dinizulu, S. M., Hedemann, E. R., Ogle, R. R., & Pasalich, D. S. (2015). Common elements of adolescent prevention programs: Minimizing burden while maximizing reach. *Administration and Policy in Mental Health and Mental Health Services Research,**42*(2), 209–219. 24504979 10.1007/s10488-014-0541-9

[CR21] Boustani, M. M., Frazier, S. L., Chu, W., Lesperance, N., Becker, K. D., Helseth, S. A., & Chorpita, B. F. (2020a). Common elements of childhood universal mental health programming. *Administration and Policy in Mental Health and Mental Health Services Research,**47*, 475–486. 32080783 10.1007/s10488-020-01023-4

[CR22] Boustani, M. M., Daleiden, E., Bernstein, A., Michelson, D., Gellatly, R., Malik, K., & Chorpita, B. (2020b). Using relevance mapping methodology to design an adolescent mental health intervention in India. *Global Health Action,**13*(1), 1775062. 10.1080/16549716.2020.1775062 32588780 10.1080/16549716.2020.1775062 PMC7480414

[CR23] Boustani, M. M., Frazier, S. L., & Lesperance, N. (2017a). Sexual health programming for vulnerable youth: Improving knowledge, attitudes, and behaviors. *Children and Youth Services Review,**73*, 375–383.

[CR24] Boustani, M. M., Gellatly, R., Westman, J. G., & Chorpita, B. F. (2017b). Advances in cognitive behavioral treatment design: Time for a glossary. *The Behavior Therapist,**40*(6), 199–208.

[CR25] Braun, V., & Clarke, V. (2006). Using thematic analysis in psychology. *Qualitative Research in Psychology,**3*(2), 77–101. 10.1191/1478088706qp063oa

[CR26] Bull, S., Thomas, D. S. K., Nyanza, E. C., & Ngallaba, S. E. (2018). Tanzania Health Information Technology (T-HIT) System: Pilot Test of a Tablet-Based System to Improve Prevention of Mother-to-Child Transmission of HIV. *JMIR mHealth and uHealth,**6*(1), e16. 10.2196/mhealth.8513 29335236 10.2196/mhealth.8513 PMC5789159

[CR27] Burke, R. J., & Richardsen, A. M. (2000). Psychological burnout in organizations: Research and intervention. In *Handbook of Organizational Behavior, Revised and Expanded* (pp. 349-386). Routledge.

[CR28] Cappella, E., Frazier, S. L., Atkins, M. S., Schoenwald, S. K., & Glisson, C. (2008). Enhancing schools’ capacity to support children in poverty: An ecological model of school-based mental health services. *Administration and Policy in Mental Health and Mental Health Services Research,**35*(5), 395. 18581225 10.1007/s10488-008-0182-y PMC3744335

[CR29] Chorpita, B. F., Daleiden, E. L., Malik, K., Gellatly, R., Boustani, M. M., Michelson, D., & Patel, V. H. (2020). Design process and protocol description for a multi-problem mental health intervention within a stepped care approach for adolescents in India. *Behaviour Research and Therapy,**133*,. 10.1016/j.brat.2020.103698 32858304 10.1016/j.brat.2020.103698

[CR30] Cohen-Katz, J., Wiley, S. D., Capuano, T., Baker, D. M., Kimmel, S., & Shapiro, S. (2005). The effects of mindfulness-based stress reduction on nurse stress and burnout, Part II: A quantitative and qualitative study. *Holistic Nursing Practice,**19*(1), 26–35. 10.1097/00004650-200501000-00008 15736727 10.1097/00004650-200501000-00008

[CR31] Coker, T. R., Elliott, M. N., Kataoka, S., Schwebel, D. C., Mrug, S., Grunbaum, J. A., Cuccaro, P., Peskin, M. F., & Schuster, M. A. (2009). Racial/Ethnic disparities in the mental health care utilization of fifth grade children. *Academic Pediatrics,**9*(2), 89–96. 10.1016/j.acap.2008.11.007 19329099 10.1016/j.acap.2008.11.007 PMC4586149

[CR32] Corrigan, P. W., McCracken, S. G., Edwards, M., Kommana, S., & Simpatico, T. (1997). Staff training to improve implementation and impact of behavioral rehabilitation programs. *Psychiatric Services,**48*(10), 1336–1338. 10.1176/ps.48.10.1336 9323757 10.1176/ps.48.10.1336

[CR34] Dedoose Version 9.0.17, cloud application for managing, analyzing, and presenting qualitative and mixed method research data (2021). Los Angeles, CA: SocioCultural Research Consultants, LLC www.dedoose.com.

[CR35] DePue, J. D., Rosen, R. K., Seiden, A., Bereolos, N., Chima, M. L., Goldstein, M. G., & McGarvey, S. T. (2013). Implementation of a culturally tailored diabetes intervention with community health workers in American Samoa. *The Diabetes educator,**39*(6), 761–771. 24052204 10.1177/0145721713504630 PMC4062972

[CR36] Ducharme, L. J., Knudsen, H. K., & Roman, P. M. (2007). Emotional exhaustion and turnover intention in human service occupations: The protective role of co-worker support. *Sociological Spectrum,**28*(1), 81–104. 10.1080/02732170701675268

[CR37] Dugani, S., Afari, H., Hirschhorn, L. R., Ratcliffe, H., Veillard, J., Martin, G., & Bitton, A. (2018). Prevalence and factors associated with burnout among frontline primary health care providers in low-and middle-income countries: A systematic review. *Gates open research,**2*, 4. 10.12688/gatesopenres.12779.3 29984356 10.12688/gatesopenres.12779.3 PMC6030396

[CR38] Dyer, K., Hooke, G. R., & Page, A. C. (2016). Effects of providing domain specific progress monitoring and feedback to therapists and patients on outcome. *Psychotherapy Research,**26*(3), 297–306. 10.1080/10503307.2014.983207 25506654 10.1080/10503307.2014.983207

[CR39] Edwards, D., Burnard, P., Coyle, D., Fothergill, A., & Hannigan, B. (2000). Stress and burnout in community mental health nursing: A review of the literature. *Journal of Psychiatric and Mental Health Nursing,**7*(1), 7–14. 10.1046/j.1365-2850.2000.00258.x 10.1046/j.1365-2850.2000.00258.x 11022506

[CR40] Edwards, D., Burnard, P., Hannigan, B., Cooper, L., Adams, J., Juggessur, T., Fothergil, A., & Coyle, D. (2006). Clinical supervision and burnout: The influence of clinical supervision for community mental health nurses. *Journal of Clinical Nursing,**15*(8), 1007–1015. 10.1111/j.1365-2702.2006.01370.x 16879545 10.1111/j.1365-2702.2006.01370.x

[CR41] Ewers, P., Bradshaw, T., McGovern, J., & Ewers, B. (2002). Does training in psychosocial interventions reduce burnout rates in forensic nurses? *Journal of Advanced Nursing*, *37*(5), 470–476. 10.1046/j.1365-2648.2002.02115.x 11843986 10.1046/j.1365-2648.2002.02115.x

[CR43] Frazier, S. L., Chou, T., Ouellette, R. R., Helseth, S. A., Kashem, E. R., & Cromer, K. D. (2019). Workforce support for urban after-school programs: Turning obstacles into opportunities. *American Journal of Community Psychology,**63*(3–4), 430–443. 31002394 10.1002/ajcp.12328 PMC7147944

[CR44] Frazier, S. L., Dinizulu, S. M., Rusch, D., Boustani, M. M., Mehta, T. G., & Reitz, K. (2015). Building resilience after school for early adolescents in urban poverty: Open trial of Leaders@ Play. *Administration and Policy in Mental Health and Mental Health Services Research,**42*, 723–736. 25425012 10.1007/s10488-014-0608-7 PMC8513122

[CR45] Fulton, B. D., Scheffler, R. M., Sparkes, S. P., Auh, E. Y., Vujicic, M., & Soucat, A. (2011). Health workforce skill mix and task shifting in low income countries: A review of recent evidence. *Human Resources for Health,**9*(1), 1. 10.1186/1478-4491-9-1 21223546 10.1186/1478-4491-9-1 PMC3027093

[CR46] Gary, F. A. (2005). Stigma: Barrier to mental health care among ethnic minorities. *Issues in mental health nursing,**26*(10), 979–999. 10.1080/01612840500280638 16283995 10.1080/01612840500280638

[CR47] Gilkey, M., Garcia, C. C., & Rush, C. (2011). Professionalization and the Experience-Based Expert: Strengthening Partnerships Between Health Educators and Community Health Workers. *Health Promotion Practice,**12*(2), 178–182. 10.1177/1524839910394175 21427271 10.1177/1524839910394175

[CR48] Glasgow, R. E., Kessler, R. S., Ory, M. G., Roby, D., Gorin, S. S., & Krist, A. (2014). Conducting rapid, relevant research: Lessons learned from the My Own Health Report project. *American Journal of Preventative Medicine,**47*(2), 212–219. 10.1016/j.amepre.2014.03.007 10.1016/j.amepre.2014.03.007 PMC4609529 24953520

[CR50] Gluschkoff, K., Elovainio, M., Kinnunen, U., Mullola, S., Hintsanen, M., Keltikangas-Järvinen, L., & Hintsa, T. (2016). Work stress, poor recovery and burnout in teachers. *Occupational Medicine,**66*(7), 564–570. 10.1093/occmed/kqw086 27412428 10.1093/occmed/kqw086

[CR53] Guerrero, A. P. S., Balon, R., Beresin, E. V., Louie, A. K., Coverdale, J. H., Brenner, A., & Roberts, L. W. (2019). Rural Mental Health Training: An Emerging Imperative to Address Health Disparities. *Academic Psychiatry,**43*(1), 1–5. 10.1007/s40596-018-1012-5 30535843 10.1007/s40596-018-1012-5

[CR54] Hannigan, B., Edwards, D., Coyle, D., Fothergill, A., & Burnard, P. (2000). Burnout in community mental health nurses: Findings from the all-Wales stress study. *Journal of Psychiatric and Mental Health Nursing,**7*(2), 127–134. 10.1046/j.1365-2850.2000.00279.x 10.1046/j.1365-2850.2000.00279.x 11146908

[CR55] Haq, Z., Iqbal, Z., & Rahman, A. (2008). Job stress among community health workers: A multi-method study from Pakistan. *International Journal of Mental Health Systems,**2*(1), 15. 10.1186/1752-4458-2-15 18954470 10.1186/1752-4458-2-15 PMC2584063

[CR56] Hedemann, E. R., & Frazier, S. L. (2017). Leveraging after-school programs to minimize risks for internalizing symptoms among urban youth: Weaving together music education and social development. *Administration and Policy in Mental Health and Mental Health Services Research,**44*, 756–770. 27544670 10.1007/s10488-016-0758-x PMC5318297

[CR133] Hennink, M., & Kaiser, B. N. (2022). Sample sizes for saturation in qualitative research: A systematic review of empirical tests. *Social Science & Medicine*, *292*, 114523. 10.1016/j.socscimed.2021.114523 10.1016/j.socscimed.2021.114523 34785096

[CR57] Hoeft, T. J., Fortney, J. C., Patel, V., & Unützer, J. (2018). Task-Sharing Approaches to Improve Mental Health Care in Rural and Other Low-Resource Settings: A Systematic Review. *Journal of Rural Health,**34*(1), 48–62. 10.1111/jrh.12229 10.1111/jrh.12229 PMC5509535 28084667

[CR59] Huang, L., Macbeth, G., Dodge, J., & Jacobstein, D. (2004). Transforming the Workforce in Children’s Mental Health. *Administration and Policy in Mental Health and Mental Health Services Research,**32*(2), 167–187. 10.1023/B:APIH.0000042745.64582.72 15586849 10.1023/b:apih.0000042745.64582.72

[CR60] Jacobs, R. H., Guo, S., Kaundinya, P., Lakind, D., Klein, J., Rusch, D., Walden, A., Mehta, T., & Atkins, M. (2017). A Pilot Study of Mindfulness Skills to Reduce Stress among a Diverse Paraprofessional Workforce. *Journal of Child and Family Studies,**26*(9), 2579–2588. 10.1007/s10826-017-0771-z

[CR62] Johnson, K. F., & Brookover, D. L. (2020). Counselors’ role in decreasing suicide in mental health professional shortage areas in the United States. *Journal of mental health counseling,**42*(2), 170–186. 10.17744/mehc.42.2.06

[CR63] Kenneth Wells, M. D., & M.P.H., Ruth Klap, Ph.D., Alan Koike, M.D., and & Cathy Sherbourne, Ph.D. (2001). Ethnic Disparities in Unmet Need for Alcoholism, Drug Abuse, and Mental Health Care. *American Journal of Psychiatry,**158*(12), 2027–2032. 10.1176/appi.ajp.158.12.2027 11729020 10.1176/appi.ajp.158.12.2027

[CR64] Kepley, H. O., & Streeter, R. A. (2018). Closing behavioral health workforce gaps: A HRSA program expanding direct mental health service access in underserved areas. *American Journal of Preventive Medicine,**54*(6), S190–S191. 29779541 10.1016/j.amepre.2018.03.006

[CR65] Kahn, R. L., Wolfe, D. M., Quinn, R. P., Snoek, J. D., & Rosenthal, R. A. (1964). *Occupational stress: Studies in role conflict and ambiguity*. John Wiley.

[CR66] Kluger, A. N., & DeNisi, A. (1996). The effects of feedback interventions on performance: A historical review, a meta-analysis, and a preliminary feedback intervention theory. *Psychological Bulletin,**119*(2), 254.

[CR67] Knifton, L. (2012). Understanding and addressing the stigma of mental illness with ethnic minority communities. *Health Sociology Review,**21*(3), 287–298. 10.5172/hesr.2012.21.3.287

[CR68] Lambert, M. J. (2012). Helping clinicians to use and learn from research-based systems: The OQ-analyst. *Psychotherapy (Chicago, Ill.),**49*(2), 109–114. 10.1037/a0027110 22642518 10.1037/a0027110

[CR69] Lambert, M. J., & Shimokawa, K. (2011). Collecting client feedback. *Psychotherapy,**48*(1), 72–79. 10.1037/a0022238 21401277 10.1037/a0022238

[CR70] Lambert, M. J., Whipple, J. L., Hawkins, E. J., Vermeersch, D. A., Nielsen, S. L., & Smart, D. W. (2003). Is It Time for Clinicians to Routinely Track Patient Outcome? A Meta-Analysis. *Clinical Psychology: Science and Practice,**10*(3), 288–301. 10.1093/clipsy.bpg025

[CR71] Lee, S., Laiewski, L., & Choi, S. (2014). Racial-ethnic variation in US mental health service use among Latino and Asian non-US citizens. *Psychiatric Services,**65*(1), 68–74. 10.1176/appi.ps.201200430 24081115 10.1176/appi.ps.201200430

[CR72] Lee, L., Montgomery, S., Gamboa-Maldonado, T., Nelson, A., & Belliard, J. C. (2019). Perceptions of organizational readiness for training and implementation of clinic-based community health workers. *Journal of Health Organizatin and Management,**33*(4), 478–487. 10.1108/jhom-06-2018-0158 10.1108/JHOM-06-2018-0158 31282813

[CR74] Li, L., Hu, H., Zhou, H., He, C., Fan, L., Liu, X., Zhang, Z., Li, H., & Sun, T. (2014). Work stress, work motivation and their effects on job satisfaction in community health workers: A cross-sectional survey in China. *British Medical Journal Open,**4*(6), e004897. 10.1136/bmjopen-2014-004897 10.1136/bmjopen-2014-004897 PMC4054641 24902730

[CR75] Lyon, A. R., & Koerner, K. (2016). User-Centered Design for Psychosocial Intervention Development and Implementation. *Clinical Psychology: Science and Practice,**23*(2), 180–200. 10.1111/cpsp.12154 29456295 10.1111/cpsp.12154 PMC5812700

[CR76] Lyon, A. R., Munson, S. A., Renn, B. N., Atkins, D. C., Pullmann, M. D., Friedman, E., & Areán, P. A. (2019). *Use of Human-Centered Design to Improve Implementation of Evidence-Based Psychotherapies in Low-Resource Communities: Protocol for Studies Applying a Framework to Assess Usability JMIR Research Protocols,**8*(10), e14990–e14990. 10.2196/14990 31599736 10.2196/14990 PMC6819011

[CR131] Malcarney, M. B., Pittman, P., Quigley, L., Horton, K., & Seiler, N. (2017). The Changing Roles of Community Health Workers. *Health Services Research*, 52 Suppl 1(Suppl 1), 360–382. 10.1111/1475-6773.12657 10.1111/1475-6773.12657 PMC5269544 28127766

[CR77] Mañas, M. A., Díaz-Fúnez, P., Pecino, V., López-Liria, R., Padilla, D., & Aguilar-Parra, J. M. (2018). Consequences of team job demands: Role ambiguity climate, affective engagement, and extra-role performance. *Frontiers in psychology,**8*, 2292. 29375424 10.3389/fpsyg.2017.02292 PMC5767326

[CR78] Martin, M. A., Perry-Bell, K., Minier, M., Glassgow, A. E., & Van Voorhees, B. W. (2018). A Real-World Community Health Worker Care Coordination Model for High-Risk Children. *Health Promotion Practice,**20*(3), 409–418. 10.1177/1524839918764893 29611433 10.1177/1524839918764893

[CR79] Maslach, C., Schaufeli, W. B., & Leiter, M. P. (2001). Job burnout. *Annual Review of Psychology,**52*, 397–422. 10.1146/annurev.psych.52.1.397 11148311 10.1146/annurev.psych.52.1.397

[CR80] McAlpine, D. D., & Mechanic, D. (2000). Utilization of specialty mental health care among persons with severe mental illness: The roles of demographics, need, insurance, and risk. *Health Services Research,**35*(1 Pt 2), 277. 10778815 PMC1089101

[CR81] McQuillin, S. D., Lyons, M. D., Becker, K. D., Hart, M. J., & Cohen, K. (2019). Strengthening and Expanding Child Services in Low Resource Communities: The Role of Task-Shifting and Just-in-Time Training. *American Journal of Community Psychology,**63*(3–4), 355–365. 10.1002/ajcp.12314 30834554 10.1002/ajcp.12314

[CR83] Merwin, E., Hinton, I., Dembling, B., & Stern, S. (2003). Shortages of rural mental health professionals. *Archives of Psychiatric Nursing,**17*(1), 42–51. 12642887 10.1053/apnu.2003.1

[CR84] Michelson, D., Malik, K., Krishna, M., Sharma, R., Mathur, S., Bhat, B., Parikh, R., Roy, K., Joshi, A., Sahu, R., Chilhate, B., Boustani, M., Cuijpers, P., Chorpita, B., Fairburn, C. G., & Patel, V. (2019). Development of a transdiagnostic, low-intensity, psychological intervention for common adolescent mental health problems in Indian secondary schools. *Behaviour Research and Therapy*, 103439. 10.1016/j.brat.2019.103439 10.1016/j.brat.2019.103439 PMC7322400 31466693

[CR85] Moate, R. M., Gnilka, P. B., West, E. M., & Bruns, K. L. (2016). Stress and Burnout Among Counselor Educators: Differences Between Adaptive Perfectionists, Maladaptive Perfectionists, and Nonperfectionists. *Journal of Counseling & Development,**94*(2), 161–171. 10.1002/jcad.12073

[CR86] Mobula, L. M., Okoye, M. T., Boulware, L. E., Carson, K. A., Marsteller, J. A., & Cooper, L. A. (2015). Cultural Competence and Perceptions of Community Health Workers’ Effectiveness for Reducing Health Care Disparities. *Journal of Primary Care & Community Health,**6*(1), 10–15. 10.1177/2150131914540917 10.1177/2150131914540917 PMC7086339 24986493

[CR87] Mongelli, F., Georgakopoulos, P., & Pato, M. T. (2020). Challenges and Opportunities to Meet the Mental Health Needs of Underserved and Disenfranchised Populations in the United States. *Focus (Am Psychiatr Publ),**18*(1), 16–24. 10.1176/appi.focus.20190028 32047393 10.1176/appi.focus.20190028 PMC7011222

[CR88] Morse, G., Salyers, M. P., Rollins, A. L., Monroe-DeVita, M., & Pfahler, C. (2012). Burnout in mental health services: A review of the problem and its remediation. *Administration and Policy in Mental Health and Mental Health Services Research,**39*, 341–352. 21533847 10.1007/s10488-011-0352-1 PMC3156844

[CR90] Nadeem, E., Lange, J. M., Edge, D., Fongwa, M., Belin, T., & Miranda, J. (2007). Does stigma keep poor young immigrant and US-born black and Latina women from seeking mental health care? *Psychiatric Services,**58*(12), 1547–1554. 18048555 10.1176/ps.2007.58.12.1547

[CR91] Najafizada, S. A. M., Bourgeault, I. L., Labonte, R., Packer, C., & Torres, S. (2015). Community health workers in Canada and other high-income countries: A scoping review and research gaps. *Canadian Journal of Public Health,**106*, e157–e164. 10.17269/CJPH.106.4747 26125243 10.17269/CJPH.106.4747 PMC6972431

[CR92] Nayar, P., Apenteng, B., Nguyen, A. T., Shaw-Sutherland, K., Ojha, D., & Deras, M. (2017). Needs Assessment for Behavioral Health Workforce: A State-Level Analysis. *The Journal of Behavioral Health Services & Research,**44*(3), 465–473. 10.1007/s11414-016-9500-4 26936627 10.1007/s11414-016-9500-4

[CR94] Newman, M. G. (2000). Recommendations for a cost-offset model of psychotherapy allocation using generalized anxiety disorder as an example. *Journal of Consulting in Clinical Psychology,**68*(4), 549–555. 10965629

[CR95] Onyett, T. P., & amp, & Matt Muijen, S. (1997). Job satisfaction and burnout among members of community mental health teams. *Journal of Mental Health,**6*(1), 55–66. 10.1080/09638239719049

[CR96] Otto, M. W., Pollack, M. H., & Maki, K. M. (2000). Empirically supported treatments for panic disorder: Costs, benefits, and stepped care. *Journal of Consulting in Clinical Psychology,**68*(4), 556–563. 10965630

[CR97] Paris, M., Jr., & Hoge, M. A. (2010). Burnout in the mental health workforce: A review. *Journal of Behavioral Health Services Research,**37*(4), 519–528. 10.1007/s11414-009-9202-2 20013066 10.1007/s11414-009-9202-2

[CR98] Park, A. L., Boustani, M. M., Saifan, D., Gellatly, R., Letamendi, A., Stanick, C., Regan, J., Perez, G., Manners, D., Reding, M. E. J., & Chorpita, B. F. (2020). Community Mental Health Professionals’ Perceptions About Engaging Underserved Populations. *Administration and Policy in Mental Health and Mental Health Services Research,**47*(3), 366–379. 10.1007/s10488-019-00994-3 31721005 10.1007/s10488-019-00994-3

[CR99] Pinedo, C., & Espinoza, L. (2018, November). Establishing a need for self-care practice in promotores/as. In *APHA's 2018 Annual Meeting & Expo (Nov. 10-Nov. 14)*. APHA.

[CR100] Pumariega, A. J., Rothe, E., & Pumariega, J. B. (2005). Mental health of immigrants and refugees. *Community mental health journal,**41*, 581–597. 10.1007/s10597-005-6363-1 16142540 10.1007/s10597-005-6363-1

[CR102] Riggs, C. G. (2001). Ask the Paraprofessionals: What Are Your Training Needs? *Teaching Exceptional Children,**33*(3), 78–83.

[CR103] Rith-Najarian, L. R. (2019). *A dissemination and implementation approach to preventing anxiety and depression in young people*. University of California.

[CR104] Rith-Najarian, L. R., Boustani, M. M., & Chorpita, B. F. (2019). A systematic review of prevention programs targeting depression, anxiety, and stress in university students. *Journal of affective disorders,**257*, 568–584. 31326690 10.1016/j.jad.2019.06.035

[CR105] Roberton, T., Kasungami, D., Guenther, T., & Hazel, E. (2016). Monitoring iCCM: A feasibility study of the indicator guide for monitoring and evaluating integrated community case management. *Health Policy and Planning,**31*(6), 759–766. 10.1093/heapol/czv129 26758538 10.1093/heapol/czv129 PMC4916319

[CR106] Rodriguez, H. P., Friedberg, M. W., Vargas-Bustamante, A., Chen, X., Martinez, A. E., & Roby, D. H. (2018). The impact of integrating medical assistants and community health workers on diabetes care management in community health centers. *BMC Health Services Research,**18*(1), 875. 10.1186/s12913-018-3710-9 30458778 10.1186/s12913-018-3710-9 PMC6247511

[CR107] Rosenthal, E. L., Brownstein, J. N., Rush, C. H., Hirsch, G. R., Willaert, A. M., Scott, J. R., Holderby, L. R., & Fox, D. J. (2010). Community Health Workers: Part Of The Solution. *Health Affairs,**29*(7), 1338–1342. 10.1377/hlthaff.2010.0081 20606185 10.1377/hlthaff.2010.0081

[CR108] Rössler, W. (2012). Stress, burnout, and job dissatisfaction in mental health workers. *European Archives of Psychiatry and Clinical Neuroscience,**262*(2), 65–69. 10.1007/s00406-012-0353-4 10.1007/s00406-012-0353-4 22926058

[CR109] Sabo, S., Allen, C. G., Sutkowi, K., & Wennerstrom, A. (2017). Community Health Workers in the United States: Challenges in Identifying, Surveying, and Supporting the Workforce. *American Journal of Public Health,**107*(12), 1964–1969. 10.2105/ajph.2017.304096 29048953 10.2105/AJPH.2017.304096 PMC5678391

[CR110] Saechao, F., Sharrock, S., Reicherter, D., Livingston, J. D., Aylward, A., Whisnant, J., & Kohli, S. (2012). Stressors and barriers to using mental health services among diverse groups of first-generation immigrants to the United States. *Community mental health journal,**48*, 98–106. 10.1007/s10597-011-9419-4 21655942 10.1007/s10597-011-9419-4

[CR112] Scarnera, P., Bosco, A., Soleti, E., & Lancioni, G. E. (2009). Preventing burnout in mental health workers at interpersonal level: An Italian pilot study. *Community Mental Health Journal,**45*(3), 222–227. 10.1007/s10597-008-9178-z 19116785 10.1007/s10597-008-9178-z

[CR114] Scott, K., Beckham, S. W., Gross, M., Pariyo, G., Rao, K. D., Cometto, G., & Perry, H. B. (2018). What do we know about community-based health worker programs? A systematic review of existing reviews on community health workers. *Human Resources for Health,**16*(1), 39. 10.1186/s12960-018-0304-x 30115074 10.1186/s12960-018-0304-x PMC6097220

[CR115] Selamu, M., Thornicroft, G., Fekadu, A., & Hanlon, C. (2017). Conceptualisation of job-related wellbeing, stress and burnout among healthcare workers in rural Ethiopia: A qualitative study. *BMC Health Services Research,**17*(1), 412. 10.1186/s12913-017-2370-5 28629360 10.1186/s12913-017-2370-5 PMC5477383

[CR116] Shernoff, E. S., Maríñez-Lora, A. M., Frazier, S. L., Jakobsons, L. J., Atkins, M. S., & Bonner, D. (2011). Teachers supporting teachers in urban schools: What iterative research designs can teach us. *School psychology review,**40*(4), 465–485. 23275682 PMC3530170

[CR119] Stalker, C., & Harvey, C. (2002). Professional Burnout in social service organizations: A review of theory, research and prevention.

[CR120] Stith, S., Pruitt, I., Dees, J. E. M. E. G., Fronce, M., Green, N., Som, A., & Linkh, D. (2006). Implementing community-based prevention programming: A review of the literature. *Journal of Primary Prevention,**27*, 599–617. 10.1007/s10935-006-0062-8 17051431 10.1007/s10935-006-0062-8

[CR121] Thomson, K. J. (2014). *Exploring the experience of community health workers operating in contexts where trauma and its exposure are continuous*. University of the Witwatersrand.

[CR122] Tijdink, J. K., Vergouwen, A. C. M., & Smulders, Y. M. (2014). Emotional exhaustion and burnout among medical professors; a nationwide survey. *BMC Medical Education,**14*(1), 183. 10.1186/1472-6920-14-183 25189761 10.1186/1472-6920-14-183 PMC4167137

[CR123] Van Dierendonck, D., Schaufeli, W. B., & Buunk, B. P. (1998). The evaluation of an individual burnout intervention program: The role of inequity and social support. *Journal of Applied Psychology,**83*(3), 392.

[CR124] van Dierendonck, D., Schaufeli, W. B., & Buunk, B. P. (2001). Burnout and inequity among human service professionals: A longitudinal study. *Journal of Occupational Health Psychology,**6*(1), 43–52. 11199256

[CR132] von Hippel, C., Brener, L., Rose, G., & von Hippel, W. (2017) Perceived inability to help is associated with client-related burnout and negative work outcomes among community mental health workers. *Health and Social Care in the Community*, *27*, 1507–1514. 10.1111/hsc.12821 10.1111/hsc.12821 31368620

[CR125] Viswanathan, M., Kraschnewski, J. L., Nishikawa, B., Morgan, L. C., Honeycutt, A. A., Thieda, P., & Jonas, D. E. (2010). Outcomes and costs of community health worker interventions: A systematic review. *Medical care,**48*(9), 792–808. 20706166 10.1097/MLR.0b013e3181e35b51

[CR128] Wennerstrom, A., Vannoy, S. D., Allen, C. E., Meyers, D., O'Toole, E., Wells, K. B., & Springgate, B. F. (2011). Community-based participatory development of a community health worker mental health outreach role to extend collaborative care in post-Katrina New Orleans. *Ethnicity & Disease*, *21*(3 0 1).https://pubmed.ncbi.nlm.nih.gov/22352080 PMC3715302 22352080

[CR129] Wilson, F. (2016). Identifying, Preventing, and Addressing Job Burnout and Vicarious Burnout for Social Work Professionals. *Journal of Evidence-Informed Social Work,**13*(5), 479–483. 10.1080/23761407.2016.1166856 27145345 10.1080/23761407.2016.1166856

[CR130] Wolpert, M., Ford, T., Trustam, E., Law, D., Deighton, J., Flannery, H., & Fugard, A. (2012). Patient-reported outcomes in child and adolescent mental health services (CAMHS): Use of idiographic and standardized measures. *Journal of Ment Health,**21*(2), 165–173. 10.3109/09638237.2012.664304 10.3109/09638237.2012.664304 22559827

