# Supplementary material for: A Qualitative Review of Community Health Workers’ Training, Supervision, and Service Delivery Needs
Source: Adm Policy Ment Health. 2025 Apr 1;52(6):1061–75. doi: 10.1007/s10488-025-01439-w (PMC12628493; doi:10.1007/s10488-025-01439-w)
Supplement: Supplementary file 2 — Supplementary file2 (PDF 165 kb) [file 10488_2025_1439_MOESM2_ESM.pdf]

Participant ID: __________Site ID: __________Date Completed: __________

Helping those who help others: Reducing stress and burnout among community health workers providing services to underserved children and families.

Semi-Structured Interview for Stakeholders and Administrators

INTRODUCTORY SCRIPT

Hello,

Thank you for agreeing to be interviewed. I know you are quite busy and I appreciate you making the time.

My name is [NAME OF INTERVIEWER], and I am a graduate student at Loma Linda University. Our research team has partnered with your program to understand yours and the CHWs needs and help create an effective training and supervision model that will support to reduce burnout and improve overall well-being. We hope our conversation today will help us to further understand any issues that you are facing in order to improve organizational support, training, and resources in your community work.

The purpose of the questions I will be asking you today is to help us learn about the CHWs current training, supervision, burnout, and any changes you wish to see.

We estimate that today's interview will take approximately 40 minutes. I will be recording our interview so that I can be a better listener and spend less time writing notes while we talk. Later, this interview will be transcribed by someone on our research team and maintained on a secure and encrypted server. This interview and its transcription will not be shared with anyone. Instead, it will be de-identified and used to create, develop, implement, and evaluate a training and supervision system (ECHOES - Empowering Community Health wOrkers with Education and Supervision). If appropriate, this system may support CHWs working in other areas (e.g. medical).

We have tried to make our questions respectful and clear. However, if you feel uncomfortable with any question, you may choose not to answer. All the questions in this interview are considered optional, and your refusal to answer questions will not impact your employment status or your eligibility to receive study compensation.

Please feel free to ask me to explain or repeat myself at any time while we are talking or after the interview. You are free to stop the interview and change your mind about participating at any time.

After completing the interview, you will receive a $25 gift card.

When the study is complete, Loma Linda Department of Psychology may submit a summary of findings to a mental health journal in the form of a research article for publication. The findings of this study are anticipated to be relevant and of great interest to mental health service researchers, providers, and community members.

Do you have any questions before we get started?

Participant ID: __________Site ID: __________Date Completed: __________

Start recording

via zoom

Interview with [PARTICIPANT ID] at [SITE ID] is being conducted by [NAME OF INTERVIEWER] on [DATE] at [TIME].

Participant ID: __________Site ID: __________Date Completed: __________

INTERVIEW

GENERAL

1. Tell me about your job.

    a. What do you enjoy about it?

    b. What is challenging about it?

This study was developed prior to the COVID-19 pandemic. As such, we are interested in learning about your stress and burnout in general. Therefore, please answer these questions thinking about your stress and burnout during regular times.

LEVELS OF BURNOUT

1. How would you describe any work-related stress that CHWs experience?

    a. Are there any main stressors you can identify?

    b. Are there any coping methods you have noticed that CHWs use?

    c. Do CHWs learn any relaxation techniques? Please tell me more about that.

CURRENT SUPERVISION AND TRAINING

Participant ID: __________Site ID: __________Date Completed: __________

1. Tell me about the training model for CHWs?
    a. What current training do they have to support the children and families they work with?
    b. How helpful do you think these training experiences have been in their day-to-day work?
    c. What strategies / intervention do you think have been most helpful?
    d. What strategies / intervention do you think have been least helpful?
    e. What gaps (if any) exist in the current training model?
    f. What can be done to improve training procedures?
2. Tell me about the CHWs current supervision model?
    a. What is helpful about the supervision meetings?
    b. What are some areas of improvement for the supervision meetings?
    c. What do you think is the goal of supervision?
3. How do CHWs know which intervention to use for what problems? Please tell me more about that.
    a. Are there problems for which there are no interventions? Which are they?
    b. Are there trainings that CHWs never need to use? Which are they?
    c. What could help you in their decision making about which intervention to use at which time?
4. How often do CHWs refer families for more intensive services?
    a. What are the main reasons they refer a family for outside services?

b. How do they know when they need to refer versus keep working with a family?

c. What could help them in their decision making about when to refer a family for more intensive services?

RECOMMENDATIONS

1. What are some tools that would be helpful for you to do a better job?

WRAP UP

Before we wrap up, is there anything that you would like to tell me that I haven't already asked about?

Thank you for taking the time to meet with me today. We value your time and opinion. Do you have any additional questions or concerns?

Give gift card.
